# Detecting positional vertigo using an ensemble of 2D convolutional neural networks^☆^

**DOI:** 10.1016/j.bspc.2021.102708

**Published:** 2021-07

**Authors:** Jacob L. Newman, John S. Phillips, Stephen J. Cox

**Affiliations:** a The School of Computing Sciences, University of East Anglia, Norwich NR4 7TJ, United Kingdom; b The Department of Ear, Nose, and Throat Surgery, Norfolk & Norwich University Hospitals NHS Foundation Trust, Norwich NR4 7UY, United Kingdom

## Abstract

The aim of the work presented here was to develop a system that can automatically identify attacks of dizziness occurring in patients suffering from positional vertigo, which occurs when sufferers move their head into certain positions. We used our novel medical device, CAVA, to record eye- and head-movement data continually for up to 30 days in patients diagnosed with a disorder called Benign Paroxysmal Positional Vertigo. Building upon our previous work, we describe a novel ensemble of five 2D Convolutional Neural Networks, using composite recognition features, including eye-movement data and three-channel accelerometer data. We achieve an F1 score of 0.63 across an 11-fold cross-fold validation experiment, demonstrating that the system can detect a few seconds of motion provoked dizziness from within over a 100 h of normal eye-movement data. We show that the system outperforms our previous 1D Neural Network approach, and that our ensemble classifier is superior to each of the individual networks it contains. We also demonstrate that our composite recognition features provide improved performance over results obtained using the individual data sources independently.

## Highlights

• We trained Deep Neural Networks to detect attacks of motion provoked dizziness.
• 2D Convolutional Deep Neural Networks outperform 1D network architectures.
• Best results were provided by input features combining eye- and head-movement.
• An ensemble of five networks outperformed each individual network alone.

## 1 Introduction

Positional vertigo is a condition whereby patients will experience a subjective sensation of spinning when moving their head into certain positions [1]. Benign Paroxysmal Positional Vertigo (BPPV) is one such cause of positional vertigo and the most common cause of dizziness [2]. The cause of dizziness in patients with BPPV lies within the semi-circular canals of the inner-ear. It is generally accepted that particles from the otolithic membrane become loose within the endolymphatic fluid and move freely in response to gravity. When patients move their head, these particles are excited within the affected canal, and the conflicting sensory information leads to a sensation of vertigo [1]. Typically, patients will report dizziness upon lying down, looking up, or turning over in bed; these triggers are consistent with the most common form of BPPV, known as posterior canalithiasis, in which the loose particles are present within the posterior canal [3].

**Fig. 1:**
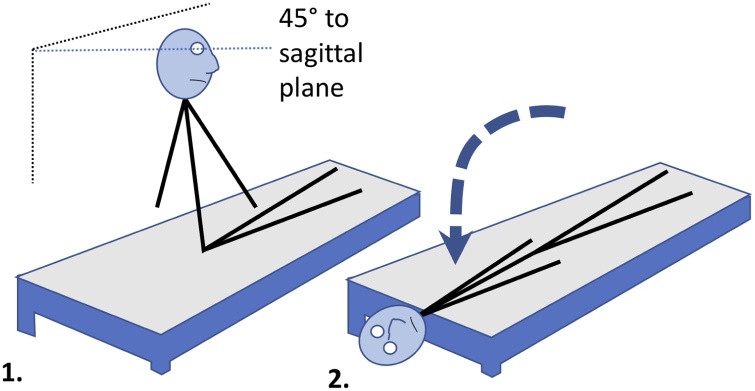
The Dix-Hallpike manoeuvre for diagnosing BPPV. (1) The patient sits on a raised couch and faces 45° away from the sagittal plane of their body. The patient faces left or right, depending on which ear is suspected of being affected by BPPV. (2) The clinician will then quickly move the patient back, maintaining the 45-degree tilt of the head, supporting the head as it hangs off the edge of the couch. Ordinarily, the patient would be instructed to keep their eyes open to allow the clinician to observe any resulting nystagmus.

The diagnosis of BPPV is relatively straightforward compared to other causes of dizziness [4]. The clinician will perform a Dix-Hallpike test, which is a simple yet optimal head manoeuvre designed to elicit a vertigo response in patients with BPPV (Fig. 1). By laying the patient back in this manner, in either a left- or right-sided test, the clinician can identify which ear is affected and subsequently administer treatment. The clinician is able to confirm the presence of vertigo by observing the patient's eyes during the test, which will show a characteristic jerking and twisting motion known as nystagmus. The Dix-Hallpike test is a very reliable way to diagnose BPPV. However, the nystagmus response can fatigue over time and the test can be performed improperly, which can stop it from being diagnosed correctly [5]. Other causes of dizziness can be more difficult to diagnose as there are no conclusive tests to establish the presence of nystagmus for vertigo that cannot be induced in a clinical setting [6]. As a result, clinicians often rely on the self-reporting of symptoms by patients [7].

**Fig. 2:**
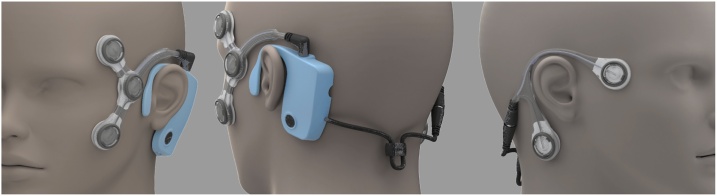
The CAVA device as worn on the face. The device includes an event marker button for the patient to highlight events of interest to the clinician, and in conjunction with the device's status LED, also provides a way for the patient to check the status of the device.

For these reasons, we have developed the Continuous Ambulatory Vestibular Assessment (CAVA) device to provide an objective record of a patient's dizziness over the course of a month. This is achieved by continuously recording eye- and head-movements by way of a small medical device worn on the face (Fig. 2). At the end of 30 days, the data captured by the device is analysed by computer algorithms for signs of nystagmus. A clinician would then review the system's findings before considering appropriate treatment options. We have previously evaluated this device in healthy volunteers with artificially induced nystagmus [8], [9], and more recently, have demonstrated that it can detect pathological nystagmus resulting from Ménière's disease and vestibular migraine [10], [11], [12], [13]. The aim of the work in this article is to develop and evaluate a system for detecting periods of nystagmus produced by patients with positional vertigo.

Our first work in this area focussed on detecting artificially induced nystagmus using an ensemble of conventional machine learning techniques including support vector machines, ensemble classifiers and decision trees [9]. We achieved good results for this task, but these approaches were less successful when we later applied them to detecting pathological nystagmus produced by Ménière's disease. Instead, we successfully applied 1D Convolutional Neural Networks (CNNs) to this task, motivated by the successful application of similar network architectures to other event classification tasks.

The nystagmus produced by Ménière's disease is different to the nystagmus produced by BPPV. Whilst nystagmus produced by Ménière's disease can persist for several hours [16], BPPV produces vertigo which typically lasts for less than a minute, often for only a few seconds [17]. Whereas nystagmus during a Ménière's attack predominantly involves the eyes moving from side-to-side, BPPV gives rise to nystagmus which is mostly present in the vertical plane [18]. The eye-movement signals captured by the CAVA device are also visibly extremely different, with nystagmus produced by Ménière's disease leading to ‘jerk’ nystagmus which has a distinctive sawtooth appearance [19], and with BPPV nystagmus appearing more oscillatory. As BPPV nystagmus is induced by movement of the head, this also raises the question of how to incorporate and combine the device's eye-movement and accelerometer data into the detection system, which was not necessary in our previous work. These are the novel challenges addressed by the work presented here.

There are several examples of work related to this topic, although none which are directly comparable. Most studies have focussed on analysing nystagmus from Videonystagmography data, which uses cameras to record eye-movement [20], [21]. By contrast, the CAVA system uses a technology similar to Electronystagmography, in which the electrophysiological signal generated by the eyeballs is recorded instead [22]. In [20], 1D CNNs were trained to discriminate between diseased and normal eye-movement signals, although this was not undertaken using continuous eye-movement data, and we are unsure as to the precise configuration of the experiments described. 2D CNNs have also been applied successfully to cardiac event detection and sleep stage classification [14], [15]. Although we do not present the results here, we have also undertaken preliminary experiments using long short-term memory networks and a variety of networks with different depths and configurations, none of which have outperformed the system described in this manuscript. In [21], peaks in the velocity signal of the eye-movements were used to identify fast phases of nystagmus. We have found such approaches to be effective when applied to isolated periods of jerk nystagmus, but not very fast or specific when applied to larger quantities of data or signals without prominent fast and slow phases, such as those examined here.

We are currently undertaking a clinical investigation of the CAVA device involving patients suffering from pathological dizziness, such as individuals with Ménière's disease, vestibular migraine and BPPV. We are in the first *training* phase of this investigation, in which patients are recruited to provide training and development data for our computer algorithms. This will be followed by a second phase in which patient data will be used as part of a blinded analysis. During the trial, patients are required to wear the CAVA device in the community, for 23 h a day, for 30 days. Thus, patients wear the device during their normal daily activities and crucially during any dizzy attacks they experience. The data used in the experiments described here are from trial participants with BPPV and also from healthy volunteers.

We have developed novel 2D CNNs capable of detecting short periods of motion-provoked dizziness, and this article describes this system in full, and presents an evaluation of its performance. The remainder of this article is organised as follows: In Section 2.1, we provide further details of the CAVA device. Then, in Section 2.2, we describe the dataset used in the experiments described here. The experimental tasks used to evaluate our system are given in Section 2.3. Section 2.4 describes the nystagmus detection system we’ve developed. The results of our experiments are given in Section 3, followed by a discussion in Section 4. The article concludes in Section 5.

## 2 Methods

### 2.1 The CAVA device

The CAVA device is intended to be worn on the face for up to a month. It continuously records horizontal and vertical eye-movement data, as well as the accelerative forces experienced by the head, in three-axes. Recording of eye-movements is achieved by way of five electrode pads which capture the electrical potential generated by the eyeballs (Fig. 2). This technology is similar to electrooculography (EOG), which has been used in clinical settings for decades to monitor eye-movements during balance assessments [23]. The CAVA device uses this technology instead of more contemporary video technology as cameras are bulky, have a larger power requirement and have implications for privacy. A further and significant reason for using EOG rather than video is that cameras cannot record eye-movements when the eyes are closed, and patients with dizziness are known to close their eyes during an attack, and attacks can start during sleep [24]. For more information about the CAVA device, please refer to our previous work [8], [9], [10].

### 2.2 The dataset

The data used in these experiments were captured using the CAVA device. The device independently captures horizontal and vertical eye-movement data at a sampling rate of approximately 42 Hz. 3-axis acceleration is sampled at approximately 20 Hz. Different combinations of these five sources of data are used in the experiments described here. The data comes from five subjects (Table 1). Subjects 1, 2 and 3 were healthy volunteers who wore the device for short periods to collect data to assist with algorithm development. Subjects 4 and 5 were participants enrolled onto our ongoing clinical investigation and who were diagnosed with BPPV. The data from Subjects 1, 2 and 3 provided a negative control for our experiments. These healthy volunteers underwent several Dix-Hallpike tests but, as they were healthy, they did not display nystagmus. These subjects performed both left-and right-sided tests.

**Table 1:** Details of data files used in our experiment. Each file has a separate ID, and contains data from one or more subjects. Also shown are the total durations of nystagmus and non-nystagmus data within each file, and whether or not that file contains negative Dix-Hallpike tests.

File ID	Subj. ID	Non-nystagmus (hh:mm:ss)	Nystagmus (hh:mm:ss)	Contains neg. Dix-Hallpikes
1	1, 2	00:16:28	00:00:00	Yes
2	3	01:48:39	00:00:00	Yes
3	3	24:01:15	00:00:00	Yes
4	4	00:23:18	00:00:25	No
5	5	24:01:15	00:00:30	No
6	5	00:08:18	00:00:19	No
7	5	00:23:32	00:00:11	No
8	5	00:28:16	00:00:31	No
9	5	24:01:03	00:00:10	No
10	5	24:00:10	00:00:19	No
11	5	24:00:45	00:00:08	No
12	5	24:01:00	00:00:13	No
13	5	24:00:39	00:00:13	No
	Total	171:34:38	00:02:59	

Subjects 4 and 5 were enrolled onto our ongoing clinical investigation, and wore the CAVA device for 30 days. Both subjects had been diagnosed with right lateral canalithiasis, meaning that they experienced vertigo when lying back with their head facing to the right. Data from Subject 4 was captured during their consenting visit for the trial, during which a clinician administered several right Dix-Hallpike head manoeuvres. Subject 5's data consisted of nystagmus captured during their consenting visit and also during the participant's 30-day trial. Hence, the majority of the data available for use in our experiments was provided by Subject 5. The nystagmus captured during the trial arose from the participant crudely and unintentionally reproducing the Dix-Hallpike manoeuvre, by moving their head into similar positions over the course of their normal daily activities.

The ground-truth labels for each subject's data were marked manually, at a sample-level. Each sample was assigned one of three possible labels: 0, 1 or 2. A label of ‘0’ meant that no nystagmus was present in the sample, ‘1’ meant that nystagmus was present, and ‘2’ meant that nystagmus was not present but the participant had placed themselves into a supine position by way of a Dix-Hallpike test. The motivation for labelling three classes was to assist our algorithms to discriminate between genuine examples of positional nystagmus and head movements in the absence of nystagmus. Labelling a positive example of nystagmus required three sources of confirmation: Firstly, if the nystagmus occurred outside of a clinical setting (i.e. while the patient was ‘on trial’), then the patient had to have activated the CAVA device's event marker to mark the attack onset. The patient also had to have noted the approximate time of the event in their trial diary. Finally, upon manual inspection, a labeller had to positively identify nystagmus at the time indicated by the event marker. In cases where the nystagmus occurred in a clinical setting, in place of the diary, the patient's eyes were physically observed to verify the presence of nystagmus. The precise sample-level labelling would likely vary depending on the individual labelling the data, and such variation reflects the subjectivity of the labelling process. However, we believe that this approach is sufficiently accurate for our purposes, to enable the presence of nystagmus to be confirmed.

### 2.3 The experiments

To evaluate the performance of our detection system, we used cross-fold validation with 11 folds (Table 2). In this approach, each fold is tested in turn, with the remaining 10 folds used as training data. Folds #1 to #3 evaluate negative control data as well as subject-independent test data. Folds #4 to #11 evaluate test data which comes solely from Subject 5. Much of this subject's data is long-term data obtained during their 30-day trial period, reflecting the way that the CAVA system will be formally evaluated at the end of our ongoing investigation. Using this testing framework, we conducted four separate experiments: (i) The first experiment was designed to determine baseline performance. We compared the performance of our new 2D CNN to the 1D CNN architecture used in our previous work. (ii and iii) We sought to determine the contribution to system performance of the eye-movement and accelerometer data. This was achieved by undertaking two separate experiments, one using eye-movement alone and another using accelerometer data alone. (iv) The final experiment was to evaluate the performance of our 2D CNN approach, including the benefits offered by our ensemble approach to classification. The network architectures used in these different experiments were broadly the same (see Section 2.4.4), except for the experiments using fewer data sources, for which the kernel sizes had to be reduced to reflect the dimensionality of the data.

**Table 2:** The data used for each fold of the cross-fold validation experiments. The IDs refer to the files listed in Table 1.

Fold #	Training file IDs	Testing file IDs	Testing subj. IDs
1	3, 4, 6, 7, 8, 9, 10, 11, 12, 13	1, 2, 5	1, 2, 3, 5
2	4, 5, 6, 7, 8, 9, 10, 11, 12, 13	3	3
3	3, 5, 6, 7, 8, 9, 10, 11, 12, 13	4	4
4	3, 4, 5, 7, 8, 9, 10, 11, 12, 13	6	5
5	3, 4, 5, 6, 8, 9, 10, 11, 12, 13	7	5
6	3, 4, 5, 6, 7, 9, 10, 11, 12, 13	8	5
7	3, 4, 5, 6, 7, 8, 10, 11, 12, 13	9	5
8	3, 4, 5, 6, 7, 8, 9, 11, 12, 13	10	5
9	3, 4, 5, 6, 7, 8, 9, 10, 12, 13	11	5
10	3, 4, 5, 6, 7, 8, 9, 10, 11, 13	12	5
11	3, 4, 5, 6, 7, 8, 9, 10, 11, 12	13	5

### 2.4 The detection system

**Fig. 3:**
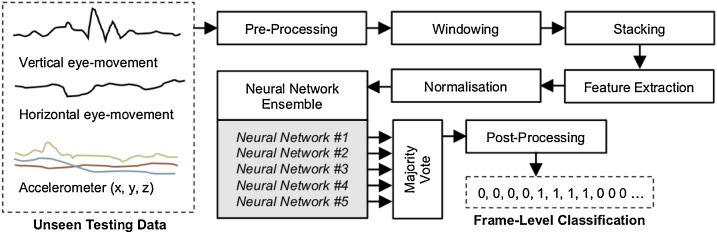
Block diagram of nystagmus detection system. Input recognition features are combined into either a 2D matrix or multi-channel 1D vector.

The system used in these experiments is a development of our previous work [10]. Fig. 3 shows the arrangement of this system and the following sub-sections describe the function of the modules within it.

#### 2.4.1 Pre-processing

Electrooculography recordings of eye-movement data commonly display signal drift, whereby the baseline of the signal moves through time. To remove this drift from our horizontal and vertical eye-movement data, we applied a second order Butterworth high-pass filter, with a cut-off frequency of 0.25 Hz. The accelerometer data was not filtered. As the CAVA device captures eye-movement and accelerometer data at different sampling rates, we linearly interpolated the accelerometer data to match the eye-movement data, allowing the vectors to be stacked into a matrix (Section 2.4.3).

#### 2.4.2 Windowing

A sliding window with a duration of 400 samples and a 75% overlap was used to convert the individual samples into separate frames of data, each representing 9.6 s of data. For training, the ground-truth label for each frame was determined by the majority label of the samples within it. During testing, a frame was considered as a positive example of ‘nystagmus’ if any of its constituent samples contained nystagmus. The reason for this difference was to ensure that only unambiguous examples of nystagmus were used for training the networks, whilst all examples of nystagmus were considered at testing. We opted for a window duration of 400 samples, based upon the findings from our previous work [10].

#### 2.4.3 Data stacking and feature extraction

There are five separate data sources available, and they are used in different combinations depending on the specific experiment undertaken. The first two sources are the eye-movement data, corresponding to the horizontal and vertical eye-movements. The last three sources are the accelerometer data channels. For this data, and consistent with our previous work, we use the eye-movement signal velocity instead of the original time-series signal. This is achieved by way of a simple differencing between adjacent samples. Although signal drift is largely removed by the high-pass filtering described in Section 2.4.1, using signal velocity also helps in this regard and makes the nystagmus more consistent between examples. Calculating the velocity reduces the signal length by one sample, and so an extra sample with a value of 0 was appended to the beginning of each vector. The five sources were then stacked to produce a feature matrix with 400 columns and 5 rows. Each row of this feature matrix was normalised to be a unit vector.

#### 2.4.4 Neural network architecture

**Fig. 4:**
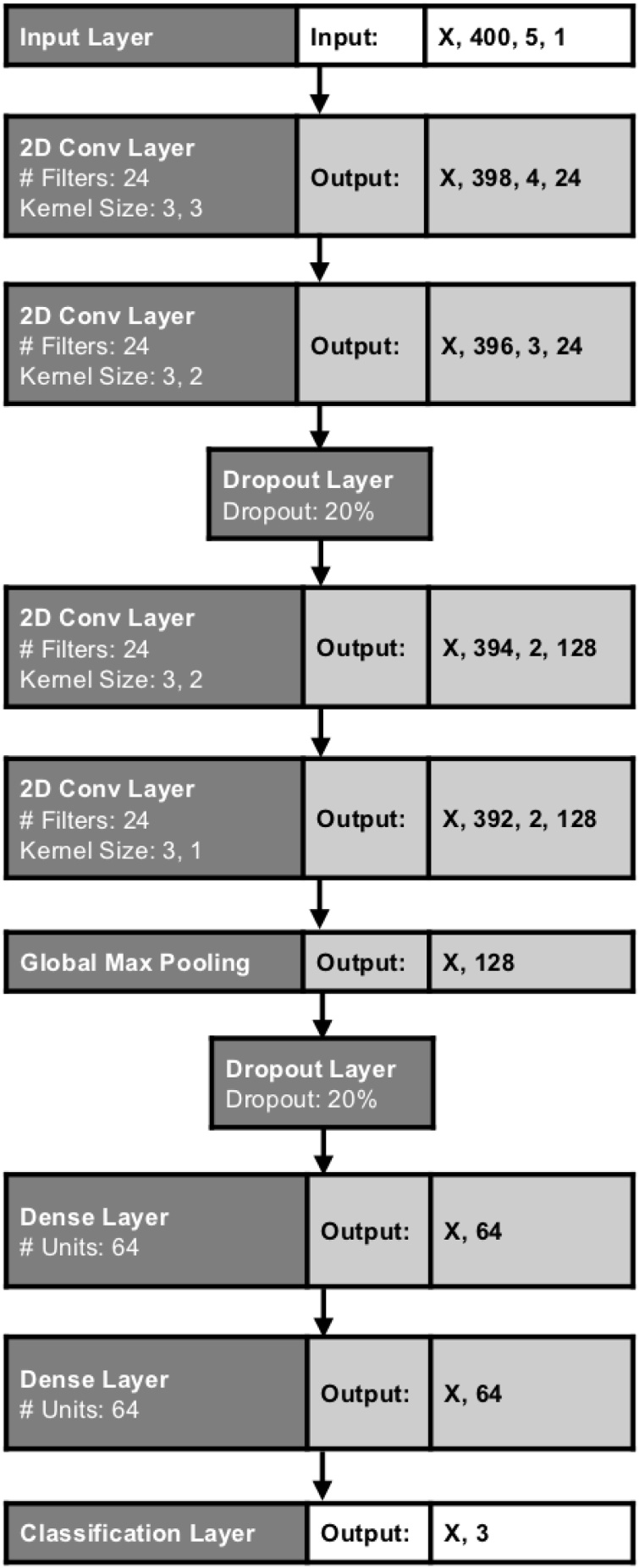
The 2D Convolutional Neural Network architecture used in our experiments. ‘X’ denotes input samples.

Fig. 4 shows the 2D CNN architecture used in our experiments. It is a development of the architecture we used in our previous work involving 1D CNNs [10]. This network differs in that it uses 2D instead of 1D convolution and the classification layer is configured for a three-class problem, rather than for two classes. The networks were trained using an Adam's optimiser, a learning rate of 0.001 and a batch size of 20. Categorical cross-entropy was the selected loss function and accuracy was the performance metric. On an Nvidia 1080 Ti GPU, each network took approximately 13 h to train to 30 epochs.

The 1D CNNs used in our previous work were shown to be well suited to classification of nystagmus occurring almost entirely in the horizontal plane. Unlike our previous work, here there are a total of five separate data channels offering potentially discriminative information (two for eye-movement and three for accelerometer data). There are broadly two approaches to incorporating this additional information into a neural network architecture. The first is to treat each data source as a separate data channel. This is the approach we use here to provide baseline performance using a 1D CNN. We also use a novel approach, which is to stack the separate data sources into a 2D feature matrix and to use a 2D CNN architecture. 2D CNNs are typically applied to image recognition tasks, or image analogues, such as signal detection using spectrograms. Here, by creating a composite feature matrix, we effectively create an image which is 400 pixels in length by 5 pixels deep. Our intention is that the 2D CNN may then learn complex relationships between the different data sources in different parts of the ‘image’, in much the same way that they are considered to do in tasks such as scene identification.

#### 2.4.5 Ensemble of CNNs

Each time a neural network is trained, it is seeded with a random value and the resulting network and its discriminative performance differs as a result. It has been shown that combining the outputs of multiple machine learning techniques can result in superior classification performance [25].

**Fig. 5:**
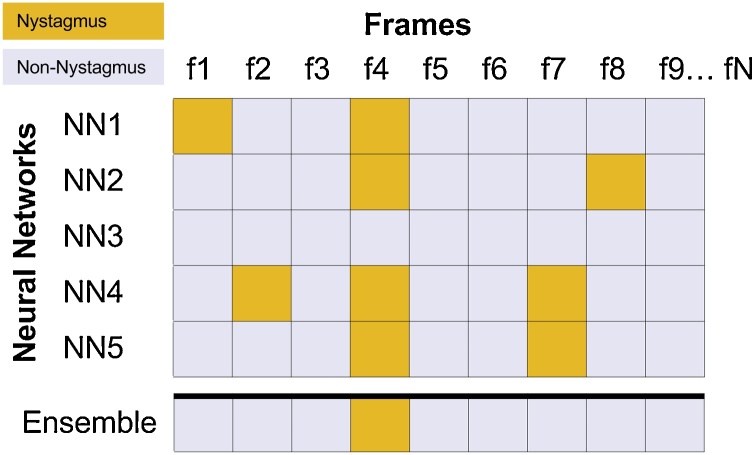
An illustration of how each network output is combined by the ensemble classifier. Networks NN1 to NN5 produce a classification for frames f1 to fN. Positive classifications shown in yellow. The Ensemble output is determined by the majority vote for each frame. In this example, NN1's positive classification of f1 does not propagate through to the Ensemble output. By contrast, four networks have classified f4 as a positive example of nystagmus, and therefore this class wins by majority vote and is present in the Ensemble output.

Here, we adopted an ensemble of five CNNs in all of the experiments undertaken. Each CNN provides its classification for each frame of data tested and the majority decision of the CNNs determines the final classification assigned to that frame (Fig. 5). Using an odd number of CNNs avoids the possibility of a tie between the classifiers. Five CNNs provide a good balance between diverse classifier outputs, and the computational time required to train and test the networks – Training five networks takes approximately three days on an Nvidia 1080 Ti. Each network is trained using labelled training data, according to the training and testing folds described in Table 2.

Table 1 shows that there is a large imbalance between the quantities of training data for the three classes considered here, with only 3 min of nystagmus data and over 171 h of non-nystagmus data. Failing to address a class imbalance such as this can result in poorly trained networks which classify everything as belonging to one class. Following our previous work, in which we showed oversampling to be an effective technique for addressing class data imbalances [10], here we applied Synthetic Minority Over-Sampling Technique (SMOTE) to our training data [26]. In summary, this technique operates by synthesising new examples of the minority class (or classes) such that the quantity of training data is equal for each class. This is achieved by interpolating the feature space between neighbouring data points. After applying this step to the training data, each of the three classes contained the same number of frames as the largest class (i.e. class ‘0’, non-nystagmus). Note that this technique is not applied to the testing data.

#### 2.4.6 Classification

Each frame was classified as one of three possible classes: 0 (non-nystagmus), 1 (nystagmus) and 2 (non-nystagmus, but with the participant in a supine position during a Dix-Hallpike test). Any frame classified as class 2 was reassigned to class 0, as the purpose of this class was only to aid the networks in discriminating between nystagmus and normal eye-movements. As with our previous work, the output from the classification stage was filtered using a sieve filter, to smooth the classification output. This filter operates by removing (or ‘opening’) short runs of positive classifications and by filling in (or ‘closing’) short runs of negative classifications [27].

## 3 Results

The results obtained by our baseline 1D CNN approach were extremely poor, caused by a low true positive detection rate of just 9% (see Exp. #1 in Table 3). The result for this experiment was even worse when including the sieve filter step, which removes short duration classifications. Similar results were produced by our new 2D CNN architecture when using only horizontal and vertical eye-movement velocity data as the recognition features (Exp. #2). Using the three-axis accelerometer data as the recognition features for the 2D CNN gave improved performance, with a sensitivity of 40%, but also with an 11-fold higher rate of false positive detections (Exp. #3). The highest performance was obtained using a 2D CNN architecture with recognition features containing both eye and head-movement data (Exp. #4).

**Table 3:** Results of a frame-level classification task for four different CNN configurations: A 1D CNN, and three experiments using a 2D CNN. All experimental results were obtained using a five-network ensemble and the same processes as described in Section 2. The 1D CNN experiment did not use the sieve filter step, as its inclusion removed all true positive detections. When calculating mean and standard error of F1 scores, we assumed a score of 1.00 for fold #2 in cases where no false positive detections were made, as the fold contains no examples of nystagmus.

Experiment #	tp	tn	fp	fn	Sensitivity (%)	Specificity (%)	F1	Mean (SE) F1
1D CNN baseline: velocity & accelerometer features								
1	13	222261	272	128	9	100	0.06	0.21 (0.09)
2D CNN: eye-movement features								
2	0	222483	50	141	0	100	0.00	0.09 (0.09)
2D CNN: accelerometer features								
3	56	221963	570	85	40	100	0.15	0.26 (0.08)
2D CNN: velocity &accelerometer features								
4	73	222515	18	68	52	100	0.63	0.63 (0.09)

tp = true positive, tn = true negative, fp = false positive, fn = false negative.

Table 4 shows the results for the five separate 2D CNNs used within the ensemble classifier which gave the results for Exp. #4 in Table 3. The performance across the five networks was fairly consistent, but with some variation in terms of true positive and false positive detections. Network #4 produced the lowest F1 score of 0.22, while Network #5 gave an F1 score of 0.61. The F1 scores for each of the individual networks were lower than for the ensemble classifier.

**Table 4:** Frame-level results for the individual networks used in the ensemble configuration to produce the results shown for Exp. #4 in Table 3.

Network #	tp	tn	fp	fn	Sensitivity (%)	Specificity (%)	F1	Mean (SE) F1
1	51	222457	76	90	36	100	0.38	0.42 (0.11)
2	50	222491	42	91	35	100	0.43	0.37 (0.11)
3	66	222469	64	75	47	100	0.49	0.50 (0.09)
4	64	222146	387	77	45	100	0.22	0.40 (0.09)
5	75	222502	31	66	53	100	0.61	0.63 (0.09)

tp = true positive, tn = true negative, fp = false positive, fn = false negative.

In Table 5 we present the performance for the individual folds of Exp. #4, shown in Table 3. Folds #1 and #2 included data from subjects undergoing Dix-Hallpike tests producing a negative result (i.e. non-nystagmus). Only one of nearly sixty negative Dix-Hallpike tests were misidentified as containing nystagmus. Fold #3 shows the result of testing data from a subject who had no other data available for use as training data. This subject-independent result showed a very high F1 score 0.76. Folds #7 to #11 tested very long data files from subject 5, each containing a day's worth of data. An F1 score of 0.89 was achieved for these folds, demonstrating a very high degree of discrimination for nystagmus captured during the subject's normal everyday activities. The F1-score for fold #8 was 0.00, as no positive classifications were made. Upon reviewing the classifications made in this fold, we found that a single true positive detection had been filtered out during the post-processing step, as it was very short in duration. The data from folds #4 to #6 were shorter in overall duration as they related to data captured during Dix-Hallpike tests administered by a clinician. The results for these folds were consistent with the results obtained using this subject's 30-day trial data.

**Table 5:** Results for the individual folds summarised in the results for Exp. #4 in Table 3.

Fold #	tp	tn	fp	fn	Sens. (%)	Spec. (%)	F1
1	3	39343	6	21	12	100	0.18
2	0	1096	0	0	-	100	-
3	16	563	3	7	70	99	0.76
4	6	196	0	10	38	100	0.55
5	5	577	3	4	56	99	0.59
6	18	693	0	5	78	100	0.88
7	8	35017	2	0	100	100	0.89
8	0	35996	0	12	0	100	0.00
9	3	36010	2	4	43	100	0.50
10	6	35016	2	3	67	100	0.71
11	8	36008	0	2	80	100	0.89

tp = true positive, tn = true negative, fp = false positive, fn = false negative.

## 4 Discussion

The results for our 2D CNN architecture using a combination of eye-movement velocity and accelerometer data provided the best classification performance across all experiments undertaken. These results showed that this system is capable of detecting relatively short durations of nystagmus, in the order of a few seconds, and from within many hours of ‘normal’ data.

The best results were achieved using feature vectors containing both eye-movement and accelerometer data, compared to results obtained using either data source independently. This accords with intuition, as either signal alone would not provide sufficient information to confirm the presence of motion-provoked nystagmus. Accelerometer data would only reveal the position of the head, rather than the presence of nystagmus, potentially leading to misclassifications every time the patient entered a supine position. This is confirmed by the relatively high number of false positive detections produced by this experiment (Table 3, Exp. #3). Eye-movement data alone gives lower performance, as the nystagmus signals are not distinctive enough by themselves to accurately be distinguished from other, normal eye-movements. It is only when nystagmus eye-movements are identified alongside supine head positioning that networks are able to accurately identify positional nystagmus.

The results in Table 5 vary across the testing folds, reflecting the variability of the signals of interest and the difficulty of the problem we are addressing. The target nystagmus signals usually occupy just a few seconds from within many days of normal eye-movements. In total, nystagmus accounts for about 0.03% of the total data used here. It can also be very challenging to identify the target signals when they are produced by imperfect vertigo attacks producing even shorter duration nystagmus, or when impacted by motion artefacts. The result for fold #8 in Table 5 is one such example, showing a very poor detection rate due to a vertigo attack with a very short duration. Likewise, it is also challenging to avoid false positive detections from within many hours of normal, yet highly variable eye-movements. Considering these points, the results presented actually reflect a remarkably high degree of accuracy. Additionally, practically speaking, a clinician would be interested in the detection of vertigo events rather than in the precise quantification of the nystagmus duration. This is because the treatment for BPPV is non-invasive and virtually risk-free, therefore favouring diagnostic sensitivity over specificity. Here, nystagmus was detected in 9 out of 10 testing folds containing nystagmus, demonstrating a high degree of sensitivity.

When comparing the performance of our baseline 1D CNN architecture to the 2D CNN, we found that the 1D CNN provided exceptionally poor performance. This result was in contrast to our previous work, where we had successfully applied 1D CNNs to the detection of horizontal jerk nystagmus. We suspect that this difference in performance has arisen from the way that the convolution filters are applied to the data sources in these networks. Although both the 1D and 2D CNNs were trained using a combination of eye-movement and accelerometer features, for the 1D network they were arranged as separate data channels, whereas for the 2D network we stacked the features in order to permit a 2D convolution of the data. It is likely that the 1D network failed to learn the relationship between the eye- and head-movement data. This explanation is supported by our experiments using the data sources independently, which showed that a 2D CNN using eye-movement data alone provided similar performance to a 1D CNN using all available channels of data.

It was reassuring to see that examples of negative Dix-Hallpike manoeuvres were almost entirely classified as negative examples of nystagmus. This result provides further confidence that the system is not simply learning the head-movements associated with laying backwards or undergoing a Dix-Hallpike test. We were also keen to ensure that the system was not simply discriminating the identity of the individuals undergoing the Dix-Hallpike test, and so testing fold #1 included negative tests from subjects who were tested in a subject-independent manner (i.e. no data from these subjects was used in the training data). No false positive detections were made for these subjects, although six false positive frames were detected overall in this fold, from Subject 3's data.

## 5 Conclusion

In this article, we have shown that nystagmus produced as a result of positional vertigo may be automatically identified within the long-term eye-movement data captured by the CAVA device. We have found that 2D CNNs offer superior performance in this regard, and that a combination of eye-movement and head-movement data are required to reliably distinguish nystagmus eye-movements from the vast range of normal eye-movements produced during a 24-hour period. The nystagmus signals detected were markedly different from the jerk nystagmus which was the focus of our previous work. While these stark differences might help to discriminate between the conditions themselves, the characteristics of BPPV nystagmus posed a greater challenge for this detection task due to its short duration and signal variability. Despite this, our 2D CNNs were able to learn the distinctive features of BPPV nystagmus and to identify it with a good degree of confidence.

The 2D CNN ensemble approach to detection was shown to offer improved performance over a single network configuration. We also found that stacking our input features to create a composite 2D feature matrix gave vastly superior results to using a 1D multi-channel architecture. While this approach may not be best suited to all detection tasks, and other configurations of 1D network may offer improved performance, this finding shows that similar techniques can provide very different results depending on how they are configured.

We tested some data in a subject-independent manner. The results obtained suggested that our findings may generalise to a larger population of unseen patients. While the performance of the detection system is very promising, our experiments were conducted using a relatively small dataset of data captured from two symptomatic patients and three healthy volunteers. Were more training data to be available, the performance of our algorithms would likely improve. In order to demonstrate the true clinical applicability of these techniques, we would have to replicate these findings on a much larger dataset, containing many more subjects. By the end of our ongoing clinical investigation, we will have collected such a dataset and will be able to further evaluate these techniques and the methods we have published previously. That new data will also provide a platform for further development of our algorithms, and the results presented here will serve as a good baseline on which we hope to improve.

Currently, the system can detect periods of nystagmus, which is clinically useful in terms of confirming that a patient is experiencing true rotatory vertigo. The characteristics of the nystagmus detected could also assist clinicians to identify the organ system responsible for a patient's vertigo. With respect to positional vertigo, the data captured by the CAVA device is sufficiently different from that of conditions such as Ménière's disease that we have had to develop separate algorithms to detect the nystagmus from these conditions. There are also other, less-common forms of positional vertigo, such as Central Positional Nystagmus (CPN) [28]. Although we have not tested patients with CPN here, down-beating nystagmus is more commonly associated with CPN, whereas the most common BPPV nystagmus is characterised by an up-beating component [29]. Of course, patients can have coexistent conditions and there is a degree of overlap between the presentation of different diseases. For these reasons, a clinician would be expected to review the output of the CAVA system in the context of a patient's other signs and test results. Following the successful completion of our current investigation, our next objective is to further develop the CAVA system to be able to diagnose the condition responsible for a patient's nystagmus. Our ultimate aim is to fully develop and deploy this system into routine medical care, to improve the speed and accuracy of diagnosing patients with suspected vertigo.
